# Quality assessment of systematic reviews of surgical treatment of cervical spine degenerative diseases: an overview

**DOI:** 10.31744/einstein_journal/2022AO6567

**Published:** 2022-04-07

**Authors:** Nelson Astur, Delio Eulalio Martins, Michel Kanas, Rodrigo Góes Medéa de Mendonça, Aaron T. Creek, Mario Lenza, Marcelo Wajchenberg

**Affiliations:** 1 Hospital Israelita Albert Einstein São Paulo SP Brazil Hospital Israelita Albert Einstein, São Paulo, SP, Brazil.; 2 Irmandade da Santa Casa de Misericórdia de São Paulo São Paulo SP Brazil Irmandade da Santa Casa de Misericórdia de São Paulo, São Paulo, SP, Brazil.; 3 Norton Leatherman Spine Center Louisville United States Norton Leatherman Spine Center, Louisville, United States.

**Keywords:** Spinal diseases, Cervical vertebrae, Chronic disease, Hernia, Intervertebral disc, Spondylosis, Orthopedic procedures

## Abstract

**Objective:**

To gather all systematic reviews of surgical treatment of degenerative cervical diseases and assess their quality, conclusions and outcomes.

**Methods:**

A literature search for systematic reviews of surgical treatment of degenerative cervical diseases was conducted. Studies should have at least one surgical procedure as an intervention. Included studies were assessed for quality through Preferred Reporting Items for Systematic Review and Meta-analysis (PRISMA) and Assessment of Multiple Systematic Reviews (AMSTAR) questionnaires. Quality of studies was rated accordingly to their final score as very poor (<30%), poor (30%-50%), fair (50%-70%), good (70%-90%), and excellent (>90%). If an article reported a conclusion addressing its primary objective with supportive statistical evidence for it, they were deemed to have an evidence-based conclusion.

**Results:**

A total of 65 systematic reviews were included. According to AMSTAR and PRISMA, 1.5% to 6.2% of studies were rated as excellent, while good studies counted for 21.5% to 47.7%. According to AMSTAR, most studies were of fair quality (46.2%), and 6.2% of very poor quality. Mean PRISMA score was 70.2%, meaning studies of good quality. For both tools, performing a meta-analysis significantly increased studies scores and quality. Cervical spondylosis studies reached highest scores among diseases analyzed. Authors stated conclusions for interventions compared in 70.7% of studies, and only two of them were not supported by statistical evidence.

**Conclusion:**

Systematic reviews of surgical treatment of cervical degenerative diseases present “fair” to “good” quality in their majority, and most of the reported conclusions are supported by statistical evidence. Including a meta-analysis significantly increases the quality of a systematic review.

## INTRODUCTION

Neck pain is a common complaint in primary care and orthopedic settings, and is often related to cervical spine conditions. Neck pain is currently the fourth leading cause for workman’s compensation claims and functional impairment in the United States.^(1)^ Every year the cost of treatment increases, especially when surgical intervention for degenerative cervical disease is involved.^(2,3)^ Common causes for surgical intervention in the cervical spine are cervical stenosis, myelopathy, spondylosis, degenerative disc disease, and facet joint arthritis.

Although conservative treatment is the primary choice of treatment for degenerative conditions of the cervical spine in the absence of neurological impairment, severity and duration of symptoms can lead the patient and provider down the path of surgical intervention. As new surgical techniques and implants are developed, scientific evidence seems to be lacking for most surgical indications. Theoretically, systematic reviews of randomized clinical trials (RCT) are on the top of the pyramid of scientific evidence, and are the source of decision-making for most spine surgeons when conducting their clinical algorithm.

However, there appears to be a substantial mismatch between the results of systematic reviews in spine surgery and daily practice outcomes. Recently, Martins et al.,^(4)^ published an overview of systematic reviews for surgical treatment of low back pain (LBP), and reported most reviews lack methodological quality and frequently state conclusions not based on statistical evidence. Authors often suggest their evidence is underpowered by the lack of clinical trials, and criticize that the ones available present methodological flaws.

## OBJECTIVE

To assess the quality of all published systematic reviews for surgical treatment of degenerative disease of the cervical spine, as well as analyze if the results of each study are supported by their methodologies.

## METHODS

### Study design

This is an overview of systematic reviews.

### Study eligibility criteria

All systematic reviews available in the queried databases containing at least one surgical treatment for any cervical spine degenerative disease as an intervention were included. Both open and minimally invasive techniques were considered. Systematic reviews comparing non-surgical treatment strategies were excluded, as well as those involving thoracic or lumbar degenerative diseases. Included studies were categorized by conditions, such as cervical disc herniation (CDH), degenerative disc disease, cervical stenosis/myelopathy, and spondylosis. Spinal injections were not considered as a surgical intervention.

### Data extraction and study quality assessment

Six reviewers independently used a standardized form to extract data. Descriptive analysis, such as journal and date of publication, cervical disease studied, surgical intervention, conflict of interests, funding, number of included studies, primary and secondary outcomes, statistics, meta-analyses, and conclusions were assessed for each study. All reviewers assessed the quality of included studies with the Preferred Reporting Items for Systematic Reviews and Meta-Analyses (PRISMA),^(5)^ and Assessment of Multiple Systematic Reviews (AMSTAR)^(6)^ tools. Both tools are validated measurement questionnaires that assess the methodological quality of systematic reviews. PRISMA is divided into domains that were also independently analyzed (title, abstract, introduction, methods, results, discussion and funding). According to a previously published article by this same group of researches,^(4)^ we classified each PRISMA item as yes, incomplete, or no, and respectively scored as 1, 0.5, or 0 points for statistical analysis purposes. Similarly, the AMSTAR tool had each item graded as yes or no and scored as 1 or 0, respectively. All reviewers were primarily trained for each item of both questionnaires and an inter- as well as intraobserver correlation reliability was assessed for both PRISMA and AMSTAR tools, through a Kappa concordance coefficient for separate items and intraclass correlation coefficient for total scores. For PRISMA, the intraclass correlation coefficient was 0.966 (95% confidence interval – 95%CI 0.889-0.990, p<0.001), which is considered very good according to Landis et al.^(7)^ For AMSTAR, an intraclass correlation coefficient of 0.912 (95%CI 0.734-0.973, p<0.001) was achieved, also considered a very good concordance. Any disagreement that might have arisen was discussed and resolved by consensus, and with an opinion of a seventh reviewer with expertise in systematic reviews. The sum of all items scored for each questionnaire was divided by its maximum possible score, to evaluate study quality as a percentage.^(4)^ Systematic reviews were classified according to AMSTAR and PRISMA percentages as follows: very poor (<30%), poor (30%-50%), fair (50%-70%), good (70%-90%), and excellent (>90%).^(4)^ We collected the following items for every systematic review: year of publication; disease, intervention, and study control; evidence-based primary outcomes through reported statistics; and outcomes reported.

### Analysis of outcomes, interventions, and diseases

We extracted data for each included study for population, intervention, control, and outcomes (PICO). Population was grouped according to the disease investigated (CDH, myelopathy, spondylosis, degenerative disc disease) and compared. Interventions and outcomes were also grouped accordingly. Two authors independently reviewed every included article to analyze the validity of the conclusion reported. We considered conclusive studies those with a valid conclusion addressing the primary outcome. If an article reported a conclusion addressing its primary objective, we searched the article for supportive statistical evidence for the conclusion. Articles meeting this criterion were deemed to have an evidence-based conclusion.

### Search strategy

After institutional review board approval (# 2742), we conducted a literature search for systematic reviews that employed at least a single surgical treatment method of degenerative cervical spine disease, through available medical databases: MEDLINE (PubMed), EMBASE (Ovid), Cochrane Database of Systematic Reviews, and the Database of Abstracts of Reviews of Effectiveness. There was no restriction to language or date. Appendix 1 demonstrates the search strategy used for MEDLINE. For other databases, we followed the same search strategy with minimal adjustments. Two investigators independently assessed all titles and abstracts to exclude duplicate articles and selected articles with potential to be included. When any disagreement was raised, a third author was consulted to solve inconsistencies. Studies from the same research group with similar intervention and analyses had only the most recent version included.

**Appendix 1:** Search strategy for MEDLINE

a
((((Zygapophyseal Joint [mh] OR Neck Pain [mh] OR cervical pain [mh] OR neck pain [tw] OR facet joint* [tw] OR cervical pain [tw] OR Intervertebral disk degeneration [mh] OR spinal stenosis [mh] OR cervical myelopathy [tw] OR myelopathy [mh] OR hernia* [tw] OR prolapse* [tw] OR extru* [tw]))) AND (((neck surgery [tw] OR Spinal fusion [mh] OR Arthrodesis [mh] OR Laminectomy [mh] Instrumentation [tw] OR Decompression, Surgical [mh] OR fusion [tw] OR in situ fusion [tw] OR interbody fusion [tw] OR disc replacement [tw] OR arthroplasty [tw])))) AND ((meta-analysis [pt] OR Systematic Reviews [tw] OR randomized controlled trial [pt] OR controlled clinical trial [pt] OR randomized controlled trials [mh] OR random allocation [mh] OR double-blind method [mh] OR single-blind method [mh] OR clinical trial [pt] OR clinical trials [mh]))

### Statistical analysis

Statistical analysis consisted of descriptive statistics including relative and absolute frequencies. Inter-observer concordance of the AMSTAR and PRISMA scores was assessed through the Gwet AC1 coeficient.^(8)^ Numerical variables were reported as medians, quartiles, boxplots and histograms. Group comparison was performed through a Mann-Whitney test for numerical variables, while categorical variables were compared through Fisher´s exact test. A Holm test was used for post-test correction. For all tests, a p value of 0.05 was considered significant. Statistics were performed with the software R 3.4.1 (R Foundation for Statistical Computing, Vienna, Austria).

## RESULTS

**Figure 1:**
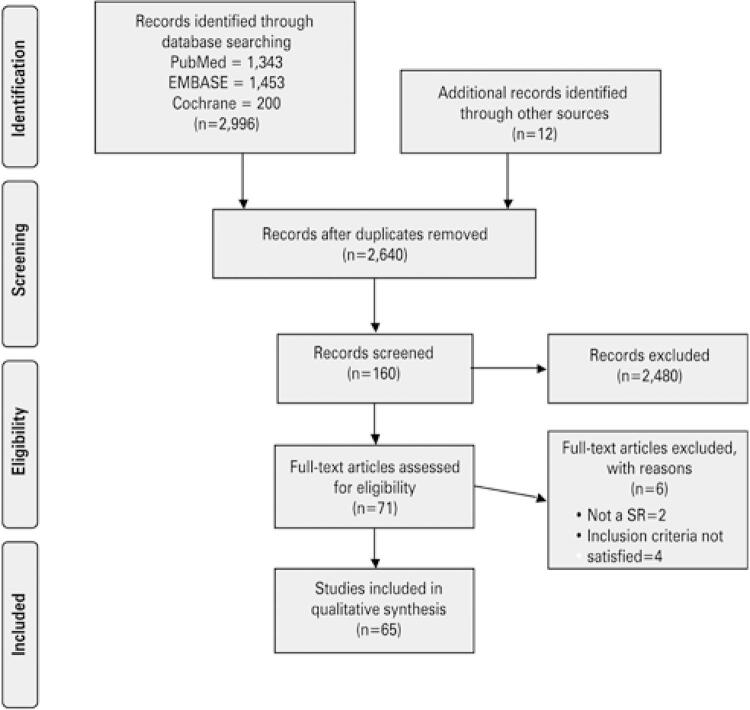
Flowchart of included articles

After a full electronic search, we identified a total of 2,996 references (Figure 1). After title, abstract and duplicate screening, we excluded 2,480 references and 71 articles were fully assessed for eligibility. Six studies were excluded (Figure 1)^(9-14)^ and 65 were included for qualitative analysis.^(15-79)^ All 65 studies were written in English, and included at least one surgical procedure to treat cervical spine degenerative disease.

SR: systematic review.

### Descriptive analysis

More than half (56.9%) of included systematic reviews of surgical treatment of cervical degenerative diseases were published within the last four years,^(15-18,20-26,29,31,35-41,43,45-48,50,53,57,58,60,62,63,65-69,73-75,77)^ with the rest ranging from 1999 to 2016. The most studied disease state was myelopathy due to degenerative compression of the spinal cord, accounting 41.5% of included studies,^(15,16,18,20-22,25,26,28,31,36,38,41,42,44,47,48,52,53,58,68-70,72,74,77)^ followed by degenerative disc disease (33.8%),^(19,23, 24,27, 30,32,39,40,43,45,49-51,57,59-62,64,73,75,78)^ spondylosis (13.8%)^(36,38, 47,57,64,66,68,72,77)^ and CDH (9.2%).^(18,34,35,55,56,67)^ There was one study that analyzed adjacent segment degeneration,^(15)^ and was not included in the pre-established groups. The surgical interventions that were most frequently analyzed were anterior cervical discectomy and fusion (ACDF) (72.3%),^(19,21,25,27,30-34,40,41,44,45,49,51,52,54,56,59,60,62-69,71,72,74,78)^ followed by total disc replacement (TDR) (44.6%).^(17,19,23-25,30,39,40,43,45,46,49,51,54,55,57,59-65,67,68,71,75,76)^ The comparison of ACDF versus TDR was considered in 27 studies (41.5%).^(19,21,25,27,31-33,40,45,49,51,52,54,56,59,60,62,64-69,71,72,74,78)^ The third most studied intervention was laminoplasty, present in 20% of included studies.^(16,17,19, 23,27,29,32,49,73,75)^

**Figure 2:**
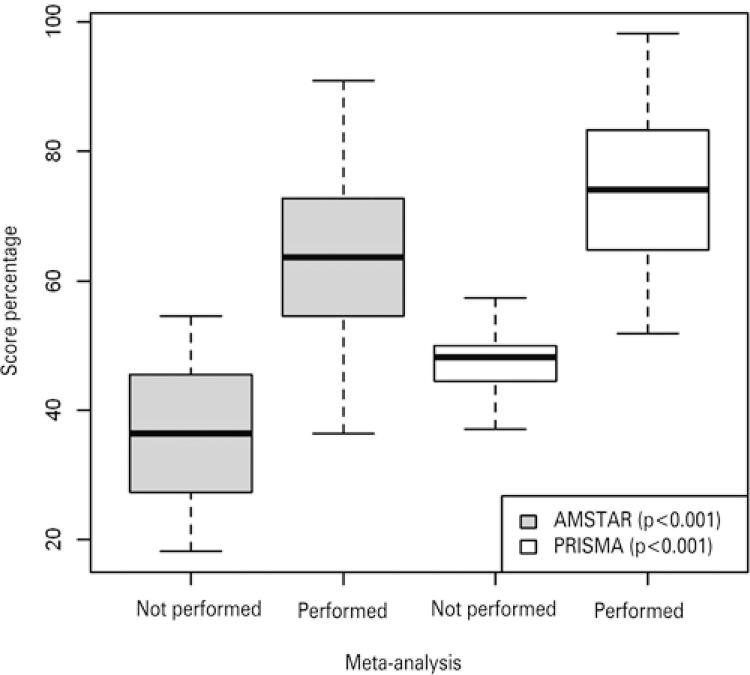
Assessment of multiple systematic reviews and Preferred Reporting Items for Systematic Reviews and Meta-Analyses percentage scores distribution, according to performing or not a meta-analysis. n=65

Most systematic reviews performed a meta-analysis (86.2%) and demonstrated statistical significance for comparison of primary outcomes in 67.7% of all included studies. Authors stated conclusions for interventions compared in 70.7% of studies. This means that two studies stated a conclusion on primary outcomes not based on statistical evidence, denoting an inconsistent conclusion.^(25,27)^ The majority of studies (56.9%) had a funding source declared other than authors own expenses, while 9.2% did not mention funding source. Potential conflicts of interest were detected in 30.8% of studies.^(15,23,24,26,28,36,38,39,41,45,47,52,53,57,60,63,64,68,78,79)^ Having or not conflicts of interest or funding declared did not influence final mean AMSTAR score (58.5% with funding versus 58.7% without funding; 58.6% with conflicts of interest versus 58.6% without conflicts). Out of the 20 articles declaring conflicts of interest, 9 analyzed TDR and 5 laminoplasty. Furthermore, 65% of those with declared conflicts of interests described methods used for assessing risk of bias, against 53.6% of those without conflicts. We compared final PRISMA and AMSTAR scores between studies with meta-analysis and those without a meta-analysis. Systematic reviews performing a meta-analysis presented significantly higher scores for both questionnaires, compared to those without (Figure 2).

The most common outcome used for surgical treatment of degenerative cervical diseases was the visual analog scale (VAS), which was present in 60% of articles, followed by the Japanese Orthopaedic Society questionnaire (JOA) and neck scale, present in 44.6% and 43.1%, respectively.

### Descriptive study quality analysis

Final percentages scored for PRISMA (total and domains) and AMSTAR are shown in table 1. Individual AMSTAR scores for each article included are shown in table 2.

**Table 1:** AMSTAR and PRISMA domains by percentage of final score

Quality tool	Mean score (%)
AMSTAR total	58.2
PRISMA total	70.2
PRISMA - Title	87.7
PRISMA - Abstract	54
PRISMA - Introduction	75
PRISMA - Methods	65
PRISMA - Results	70
PRISMA - Discussion	88.9
PRISMA - Funding	67.7

PRISMA: Preferred Reporting Items for Systematic Reviews and Meta-Analyses.

**Table 2:** Individual AMSTAR scores for each item (first row) of each article (first column). Total checked results are presented in percentage (last column) and checked items are presented as “X”

Authors	1	2	3	4	5	6	7	8	9	10	11	Total (%)
Boselie et al.,^(61)^		X	X	X	X	X	X	X	X	X	X	90.91
Zhong et al.,^(63)^		X	X	X		X	X	X	X	X	X	81.82
Bartels et al.,^(18)^	X	X	X	X		X	X	X	X		X	81.82
Li et al.,^(77)^	X	X	X			X	X	X	X	X	X	81.82
Dong et al.,^(38)^	X	X	X	X		X	X	X	X		X	81.82
Liu et al.,^(16)^		X	X	X		X	X	X	X	X	X	81.82
Luo et al.,^(34)^	X	X	X			X	X	X	X		X	72.73
Liu et al.,^(32)^		X	X			X	X	X	X	X	X	72.73
Wu et al.,^(62)^	X	X	X			X	X	X	X		X	72.73
Guan et al.,^(37)^		X	X			X	X	X	X	X	X	72.73
Lu et al.,^(60)^		X	X			X	X	X	X	X	X	72.73
Jacobs et al.,^(51)^		X	X	X		X	X	X	X		X	72.73
Botelho et al.,^(26)^		X	X		X	X	X	X	X		X	72.73
Verhagen et al.,^(20)^		X	X			X	X	X	X	X	X	72.73
Gu et al.,^(49)^	X	X	X			X	X	X	X		X	72.73
Han et al.,^(45)^	X	X	X			X	X	X	X			63.64
Rao et al.,^(64)^		X	X				X	X	X	X	X	63.64
Yin et al.,^(50)^		X	X				X	X	X	X	X	63.64
Zhang et al.,^(33)^		X	X				X	X	X	X	X	63.64
Zhang et al.,^(58)^		X	X			X	X	X	X		X	63.64
Hu et al.,^(66)^		X	X		X	X	X	X	X			63.64
Gao et al.,^(52)^		X			X	X	X		X	X	X	63.64
Xiao et al.,^(70)^		X	X			X	X	X	X		X	63.64
Luo et al.,^(43)^	X		X			X	X	X	X		X	63.64
Liu et al.,^(30)^	X		X	X			X	X	X		X	63.64
Lee et al.,^(53)^		X	X				X	X	X	X	X	63.64
van Middelkoop et al.,^(79)^		X	X				X	X	X	X	X	63.64
Wang et al.,^(73)^		X	X			X	X	X	X		X	63.64
Wang et al.,^(29)^	X	X	X				X	X	X		X	63.64
Verma et al.,^(69)^	X	X	X				X	X	X		X	63.64
Gao et al.,^(24)^	X	X	X				X	X	X		X	63.64
Liu et al.,^(48)^		X	X				X	X	X	X	X	63.64
Fei et al.,^(22)^		X	X				X	X	X	X	X	63.64
Shamji et al.,^(21)^		X	X			X	X	X	X	X		63.64
Yuan et al.,^(19)^	X	X	X			X	X	X	X			63.64
van Middelkoop et al.,^(57)^		X	X				X	X	X	X	X	63.64
Wang et al.,^(71)^	X	X	X				X	X	X	X		63.64
Yang et al.,^(68)^	X	X	X				X	X	X			54.55
Li et al.,^(74)^		X	X			X		X	X		X	54.55
Tian et al.,^(40)^		X	X	X			X	X	X			54.55
Aragones et al.,^(56)^	X		X			X	X	X	X			54.55
Fouyas et al.,^(36)^	X	X	X						X	X	X	54.55
Harrod et al.,^(25)^	X		X				X	X	X		X	54.55
Liu et al.,^(23)^		X	X	X		X	X	X				54.55
Wen et al.,^(78)^		X	X				X	X	X	X		54.55
Liu et al.,^(75)^		X	X				X	X	X			45.45
Muheremu et al.,^(72)^		X	X					X	X		X	45.45
Yu et al.,^(55)^			X				X	X	X	X		45.45
Xing et al.^(76)^				X		X			X	X	X	45.45
Fallah et al.,^(47)^	X		X				X	X	X			45.45
Zhu et al.,^(39)^		X	X					X	X		X	45.45
Cepoiu-Martin et al.,^(46)^		X	X			X	X	X				45.45
Jiang et al.,^(44)^	X	X	X				X		X			45.45
Ren et al.,^(65)^		X	X						X	X	X	45.45
Zhao et al.,^(41)^		X	X				X	X	X			45.45
Sun et al.,^(54)^		X	X						X	X	X	45.45
Lu et al.,^(60)^		X	X				X	X		X		45.45
Huang et al.,^(42)^						X	X	X	X			36.36
Shriver et al.,^(31)^		X				X	X		X			36.36
van Limbeek et al.,^(28)^		X	X				X	X				36.36
Yoon et al.,^(17)^			X	X	X	X						36.36
Gebremariam et al.,^(67)^		X					X	X				27.27
Lao et al.,^(27)^		X				X					X	27.27
Carrier et al.,^(15)^		X				X					X	27.27
Molinari et al.,^(35)^						X		X				18.18
Mean (%)	30.7	84.6	87.7	16.9	7.7	53.8	83	84.6	86.1	40	64.6	58.2

Mean total percentage for all included systematic reviews according to PRISMA was 70.2% (range 37-98.2%), demonstrating studies averaged good quality. Mean total percentage according to AMSTAR was 58.2% (range 18.2-90.9%), showing these were reviews of fair quality. Preferred Reporting Items for Systematic Reviews and Meta-Analyses domains showed variable mean scoring from 54% (abstract) up to 88.9% (discussion). Most flaws were found in the abstract (54%), methods (65%), and funding statement (67.7%), while best rates were in the discussion (88.9%), title (87.7%), and introduction (75%). Results were “good” in 70% of studies.

The items with most flaws in the PRISMA checklist were reporting risk of bias across studies and description of additional analyses, which were reported in only 32.3% and 38.5% of studies, respectively. According to AMSTAR, most reporting flaws were in providing a list of studies (included and excluded), and the item “was the status of publication used as an inclusion criterion?”.

According to PRISMA, the majority (53.9%) of studies supporting surgical treatment of degenerative cervical diseases are rated as having a “good or excellent” quality. There were no studies rated as “very poor” quality (Table 3).

**Table 3:** AMSTAR and PRISMA quality distribution by percentage of final score

Quality tool	Categories				
	Very poor (%)	Poor (%)	Fair (%)	Good (%)	Excellent (%)
AMSTAR	6.2	24.6	46.2	21.5	1.5
PRISMA	0	10.8	35.4	47.7	6.2

AMSTAR: Assessment of multiple systematic reviews; PRISMA: Preferred Reporting Items for Systematic Reviews and Meta-Analyses.

Assessment of multiple systematic reviews had more reviews rated as “fair” (46.2%), and fewer rated as “excellent” and “good” (1.5% and 21.5%, respectively). There was a considerable amount of “very poor quality” reviews (Table 2).

### Quality analysis according to disease studied

When reviews were grouped according to the disease studied, the percentage of “good” or “excellent” studies considering PRISMA was higher for spondylosis studies, achieving all nine reviews for this disease (100%),^(36,38,47,57,64,66,68,72,77)^ followed by studies of myelopathy (51.8%) and degenerative disc disease (50%). The AMSTAR tool resulted in smaller percentages of “good” or “excellent” studies, with the highest being CDH (33.3%), followed by degenerative disc disease (22.7%), myelopathy (22.2%) and spondylosis (22.2%) (Table 4).

**Table 4:** Systematic reviews quality according to the disease studied

Disease population	n	PRISMA >70% n (%)	AMSTAR >70% n (%)	PRISMA and AMSTAR >70% n (%)
CDH	6	1 (16.7)	2 (33.3)	1 (16.7)
Myelopathy	27	14 (51.8)	6 (22.2)	2 (7.4)
Spondylosis	9	9 (100)	2 (22.2)	2 (22.2)
DDD	22	11 (50)	5 (22.7)	5 (22.7)
ASD	1	0	0	0
p value		p=0.006	p=0.692	p=0.689

n=65. CDH: cervical disc herniation; DDD: degenerative disc disease; ASD: adjacent segment degeneration.

### Agreement between AMSTAR and PRISMA

**Figure 3:**
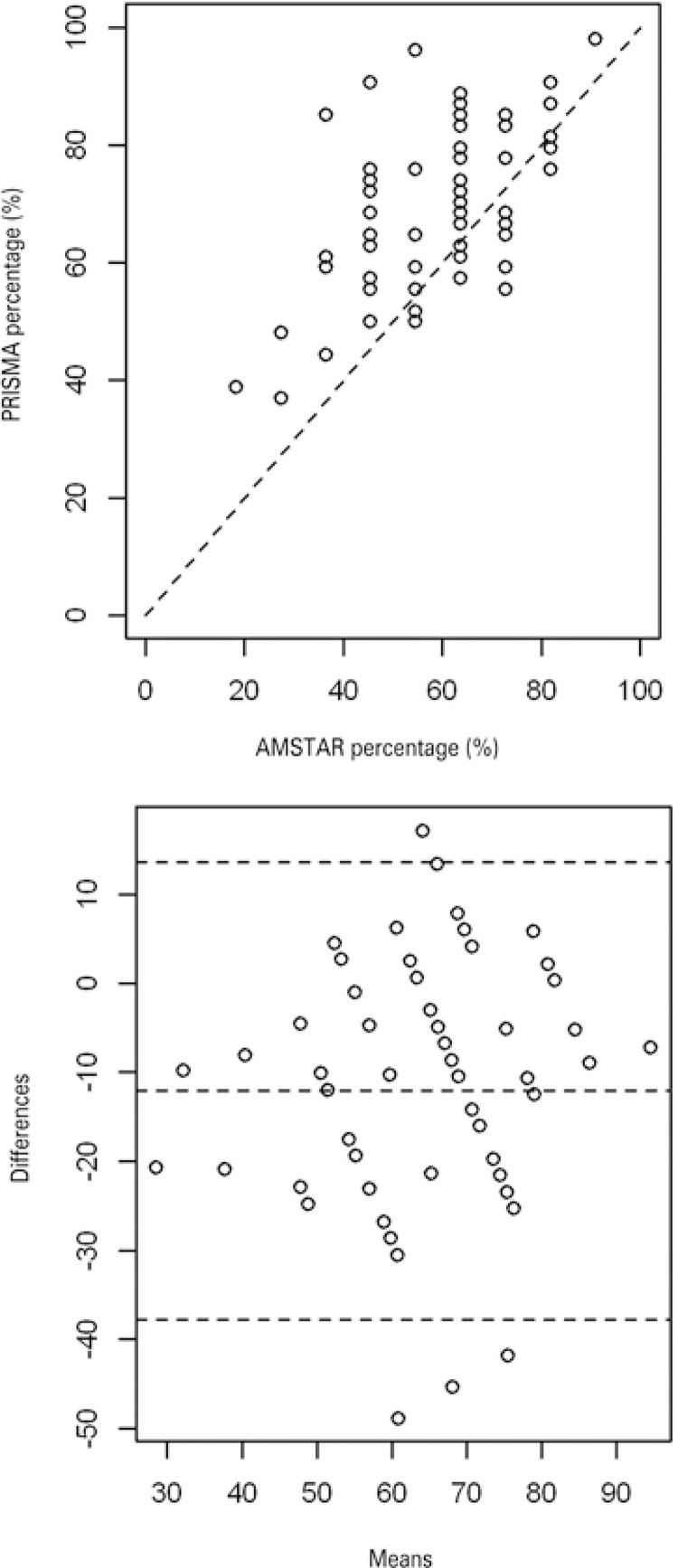
Agreement between AMSTAR and PRISMA tools according to their percentage scores. n=65

In general, PRISMA scores were higher than AMSTAR. Median difference between both tools was -10.44, although differences were randomly distributed, indicating absence of bias (Figure 3). As AMSTAR scores increase, PRISMA also rises, demonstrating concordance between both tools (intraclass correlation coefficient (ICC) 3 = 0.6, p<0.001).

## DISCUSSION

On the top of the pyramid of evidence stands systematic reviews, but frequently they do not support our decisions as they often lack quality or fail in their methodology.^(4)^ Assessment of Multiple Systematic Reviews and PRISMA are both validated checklists, which guide the production of systematic reviews. While AMSTAR focus on methodological aspects of the research, PRISMA is a more complete checklist involving all the written presentation of the published article. Although our scoring system weights the same numerical point for items inside different domains of PRISMA, such as title or methods, the weights are leveled at the total score, since there are 12 items for methods and only one for title for instance. Thus, the importance of a good-reported methodology is much higher than the paper title itself. It is expected that PRISMA reach higher scores than AMSTAR in our comparison, since there are additional items not related to the methodology of the review that are usually checked, such as those in the title and discussion domains.

In January 2016, our Study Group reported on a low rate of “good” or “excellent” quality systematic reviews for surgical treatment of LBP.^(4)^ In this study we found almost double the number of articles for cervical disease as compared to those for LBP. This is most likely because surgical treatment of cervical degenerative diseases is more standardized and less controversial than for LBP. Sixty percent of studies we reviewed for this paper were written within the last four years, while the rate was 50% for LBP. The oldest study included in the lumbar spine review was from 1992.^(80)^ The more recent systematic reviews usually follow standardized patterns and protocols before they are performed, such as Cochrane and PRISMA itself. This leads to strictly performed systematic reviews and increases methodological quality, although inclusion of selected RCTs is more exclusive, and frequently systematic reviews will not perform a meta-analysis. This might be one of the reasons we found more “good” or “excellent” articles (53.9% according to PRISMA and 25% for the LBP study)^(4)^ in the cervical spine study.

One of the developing technologies in cervical spine surgery is TDR. Much of them were performed by the need to introduce an alternate option to ACDF, and industry funding was an important resource. Funding was declared for 57% of reviews, and we identified a possible conflict of interest between authors and sponsoring industry in 30.8% of them. Sometimes conflicts of interest are disclosed to journal editors, but authors fail to report it within the manuscript will lead to lower AMSTAR and PRISMA scores, and readers might question bias. The interest in introducing a new technique is mostly originated by the implant companies, and comparison is mostly made with what is considered the gold standard. Total disc replacement was compared to ACDF in 27 (41.5%) reviews,^(19,21,25,27,31-33,40,45,49,51,52,54,56,59,60,62,64-69,71,72,74,78)^ and 12 of them concluded that TDR is superior to ACDF.^(21,31,40,49,54,60,62,65-69)^ Eleven stated that there is no superiority between both techniques,^(19,27,45,51,52,59,64,71,72,74,78)^while two reviews did not state a conclusion due to lack of studies.^(25,26)^ One review did not recommend TDR due to its high cost, and lack of proved superiority compared to ACDF.^(18)^ Only one study concluded that ACDF is superior to TDR for surgical parameters, such as shorter duration of surgery and less blood loss.^(52)^ An interesting fact that demonstrates how time, surgeon learning curve, technique accessibility, and development of good quality clinical trials, is that out of the 15 systematic reviews comparing ACDF and TDR published before 2014, only two reported a superiority of TDR, while 10 out of 12 studies published after 2014 favored TDR. Probably the publication of better clinical trials involving ACDF and TDR changed the results of most systematic reviews. Still, it is a highly controversial topic and theme of discussion in most spine surgery meetings. Theoretically, similar systematic reviews performed within the same period of time should present similar results, although we identified different conclusions. This increases the suspicion that not all reviews have the same quality, and methodology should always be double-checked. In a recent publication of quality analysis of systematic reviews comparing ACDF and TDR using only AMSTAR,^(81)^ 33.3% of reviews were considered low-quality reviews (AMSTAR <5).

Another source of debate in cervical spine surgery is posterior decompression of the spinal canal. Laminectomy *versus* laminoplasty are the most compared surgeries for cervical myelopathy in the Asia Pacific region. Laminectomy is usually the treatment choice in the United States and Europe, since the majority of spine surgeons do not perform laminoplasty. This debate is easily demonstrated, since all six systematic reviews included comparing ACDF *versus* TDR demonstrated no superiority of one technique over the other.^(17,27,29,32,49,53)^ All those six reviews were published after 2013, demonstrating recent data.

In general, the included systematic reviews completed 70.2% of the 27 items from PRISMA and 58.2% from AMSTAR, which resulted in good quality reviews according to PRISMA, and fair according to AMSTAR. From another point of view, 53.9% of systematic reviews for surgical treatment of the degenerative cervical spine were rated as “good” or “excellent” studies, while this number decreases to 23% according to AMSTAR. Although rating was different, statistical analysis demonstrated correlation between both quality tools. The reviews for cervical degenerative disease were significantly better than those for surgical treatment of LBP, that presented 25% and 32.5% of “good” or “excellent” quality studies according to PRISMA and AMSTAR, respectively.^(4)^ As stated before, this should be related to more recent reviews performed following standard protocols, and a higher number of good quality trials available in the literature.

One of the most surprising results was a high rate of conclusions supported by statistical evidence, not only by authors’ own impression. Thus, 67.7% of reviews that presented a conclusion for their primary objective were supported by demonstrated statistics, and only two papers concluded their hypotheses without support.^(23,68)^ On the other hand, only 27.5% of conclusions of the lumbar spine systematic reviews quality-study were supported by evidence.^(4)^

There are some strengths and limitations to our overview. One of the limitations is that we did not access quality of included RCT for each systematic review, which leave us with quality of the systematic reviews and not strength of recommendations, although this was not the aim of this overview. We did not contact the authors of the studies included to clarify inconsistent aspects in their reports. The high number of articles included lengthened the study period and new systematic reviews may have been published during it. The PRISMA tool itself has some items that are not essential for a correct methodology, such as the item regarding “description of additional analysis”, which is optional and not necessary. Scoring this item or not changes the result of the final score and quality of the article, although only few included systematic review performed additional analysis. On the other hand, we ran statistical correlation test to validate every author, when analyzing each systematic reviews for AMSTAR and PRISMA, and this same group of authors have previous experience with this type of assessment, as published in a similar previous overview. We searched for systematic reviews in every main database and found a significant number of included systematic reviews. Also, this is the first assessment of quality systematic reviews for surgical treatment of degenerative cervical diseases using validated tools published as far as we know.

Concerning future recommendations based on our results, we obligate the essentiality of standard guidelines, such as Cochrane Collaboration,^(82)^ to be followed before performing a systematic review or meta-analysis. The same tools used for quality assessment in this research are recommended as guidelines to perform a good quality review, such as the PRISMA statement. An overall impression due to weak results of a substantial number of systematic reviews for surgical treatment is that we still lack good clinical trials to evidence most of our surgical practice, and research teams should spend more time and energy performing trials than systematic reviews that lacks such data. On the other hand, we understand that clinical trials involving surgery are cumbersome, costly, take a long period of time and follow-up, and present numerous bias that frequently turns them unfeasible or weak. Furthermore, the primary interest of the industry is to study innovative interventions rather than repeating previous studies, even if such previous trials are of doubtful quality. Still, a significant funding for surgical trials and research is still coming from the industry, and its interest is most often conflicting.

Researchers investing their time in systematic reviews should optimize their studies into network systematic reviews that are updated regularly, ideally revised with additional new evidence included every 2 to 5 years. This would save time of doing unnecessary repeated search and analysis work. Therefore, authors suggest that readers should analyze cautiously strength of every review based on their methodology and included studies, since two good quality papers comparing same interventions may present opposing recommendations.

## CONCLUSION

Systematic reviews of surgical treatment of cervical degenerative diseases present “fair” to “good” quality in their majority, and most of the reported conclusions are supported by statistical evidence. Methodologies of studies reached average “fair” quality according to both tools. Including a meta-analysis significantly increases the quality of a systematic review.
